# Holocentric chromosome evolution in kissing bugs (Hemiptera: Reduviidae: Triatominae): diversification of repeated sequences

**DOI:** 10.1186/s13071-017-2349-4

**Published:** 2017-09-06

**Authors:** Sebastián Pita, Pedro Lorite, Jesús Vela, Pablo Mora, Teresa Palomeque, Khoa Pham Thi, Francisco Panzera

**Affiliations:** 1 Sección Genética Evolutiva, Facultad de Ciencias, Universidad de la República, Calle Iguá 4225, 11400 Montevideo, Uruguay; 2 Departamento de Biología Experimental, Área de Genética, Universidad de Jaén, Paraje Lagunillas s/n, 23071 Jaén, Spain; 3 Center for Molecular Biology, IRD, Duytan University, Danang, Vietnam

**Keywords:** Chagas disease vectors, Genomic in situ hybridization, Holocentric chromosomes, Triatominae

## Abstract

**Background:**

The analysis of the chromosomal and genome evolution in organisms with holocentric chromosomes is restricted by the lack of primary constriction or centromere. An interesting group is the hemipteran subfamily Triatominae, vectors of Chagas disease, which affects around 6 to 7 million people worldwide. This group exhibits extensive variability in the number and chromosomal location of repeated sequences such as heterochromatin and ribosomal genes. This paper tries to reveal the significant differences of the repeated sequences among *Triatoma* species through the use of genomic DNA probes.

**Methods:**

We analysed the chromosomal distribution and evolution of repeated sequences in *Triatoma* species by genomic in situ hybridization (GISH) using genomic DNA probes from two North American *Triatoma* species. These genomic probes were hybridized both on their own chromosomes and on other *Triatoma* species from North and South America, with different amounts and chromosome location of C-heterochromatin. The results were compared with those previously described using South American *Triatoma* genomic probes.

**Results:**

We observed two chromosomal hybridization patterns: (i) very intense hybridization signals concentrated on specific chromosomal regions or particular chromosomes; and (ii) lower intensity hybridization signals dispersed along all chromosomes. Self-GISH on *T. rubrofasciata* and *T. dimidiata* chromosomes presented strong hybridization signals on all C-heterochromatin regions. However, when we perform genomic cross-hybridizations, only strong signals are detected on the Y chromosome, leaving the C-heterochromatic autosomal regions unmarked.

**Conclusions:**

We confirm that repeated DNA of the Y chromosome is shared among *Triatoma* species and probably represents an ancestral character of the Triatomini tribe. On the contrary, autosomal heterochromatic regions are constituted by species-specific DNA repeats, most probably satDNA families, suggesting that *Triatoma* speciation involved the amplification of diverse types of autosomal repeats. Molecular characterization of principal repetitive DNAs seems to be an appropriate approach to infer evolutionary relationships in triatomines.

## Background

Repetitive DNA sequences consist of a large portion of eukaryotic genomes including tandem and dispersed repeats [1, 2]. Tandem repeats are organized in arrays in which the monomers (or repeat units) are repeated in a head-to-tail fashion, including multigene families (ribosomal DNAs, histone genes and small nuclear DNA) and satellite DNA (satDNA). Dispersed repeats are mainly represented by transposable elements, which are DNA segments able to change from one locus to another within the genome of their host [3].

Repetitive DNA fractions are usually larger than the coding sequence component of a genome and are essential for different genomic functions such as replication, transcription and expression [4]. For example in *Drosophila,* the important role that heterochromatin plays in the chromosome pairing, gene silencing via position-effect variegation and maintaining genome stability is well documented [5]. The chromosomal distribution of repeated DNA sequences, mainly located in the heterochromatic regions, confers a specific nuclear architecture, which establishes distinct transmission and expression characteristics even with the same coding sequences [4]. Hence, alterations in the location and composition of repeated DNA would have a diversification role in the evolution of the species [6], such as it was observed in the reproductive isolation of *Drosophila* sister species [7]. Evolutionary analyses of these sequences are even more significant in organisms with holocentric chromosomes, where karyotypes generally lack appropriate chromosomal markers for this type of study. In insects, different orders that include species with significant economic (agricultural pests) and medical importance (vectors of human diseases) exhibit this type of chromosomes. These orders are Dermaptera (earwigs), Hemiptera (true bugs), Lepidoptera (butterflies and moths), Odonata (dragonflies and damselflies), Phthiraptera (lice), Psocoptera (book lice), Zoraptera (angel insects) and Trichoptera (cloth moths) [8]. In the Hemiptera, the reduviid subfamily Triatominae comprises vectors for Chagas disease, an anthropozoonotic illness caused by the protozoan parasite *Trypanosoma cruzi*, which affects six to seven million people worldwide, mostly in Latin America but is increasingly detected in USA, Canada, and many European countries [9]. In this hemipteran group, the lack of primary constriction and their small chromosome size greatly hamper chromosome studies. Although their holokinetic structure is expected to facilitate karyotype evolution through fusions and chromosome fissions, the number of autosomes in triatomines appears to be quite stable: almost all species have a diploid autosomal number of 20 [10]. In spite of this extensive uniformity in their autosomal number, the subfamily Triatominae exhibits a great variability of the genome size [11], in the ribosomal clusters chromosome location [12–14] and in the amount, distribution and composition of the repetitive sequences included in the constitutive heterochromatin [10, 15]. Nevertheless, unlike other insects or even other hemipteran families such as Coreidae or Pentatomidae, FISH analyses using other multigene families’ probes such as 5S rDNA, U2 snDNA or histone genes, failed to achieve satisfactory results in this insect group. In this way, different methodological approaches were applied to analyse repetitive sequences of triatomines such as genomic in situ hybridization (GISH) [16] and chromosomal microdissection [17]. In insects with holocentric chromosomes, GISH approaches are scarce and have been applied to the study of sex chromosomes evolution in the Lepidoptera (reviewed by [18]) and Hemiptera (Pyrrhocoridae) [19]. A previous GISH study using genomic probes of *Triatoma* species from South America revealed that closely related species share their repetitive sequences. Furthermore, all Triatomini tribe species, including *Triatoma* and other genera, always have a Y chromosome with similar highly repeated sequences [16]. To further analyse the evolution of the repeated sequences both in autosomes and sex chromosomes in the genus *Triatoma*, in this paper we applied genomic probes from two North American *Triatoma* species. These DNA probes were hybridized both on their own chromosomes (self-GISH) and on other *Triatoma* species, with different amount and chromosomal location of the C-heterochromatin.

## Methods

The subfamily Triatominae includes 151 species in 15 genera, being the genus *Triatoma* (Triatomini tribe) the most numerous and diverse (84 species) and the only one found in the New and Old World [20]. We studied species included in two of the three main groups of this genus: (i) the rubrofasciata group (from Central and North America and Old World species): *T. barberi, T. dimidiata, T. lecticularia, T. nitida* and *T. rubrofasciata*; and (ii) the infestans group (from South America): *T. infestans* and *T.* (*Mepraia*) *spinolai*. All these species are important vectors of Chagas disease due to their presence in domestic and peridomestic environments, excepting *T. lecticularia* which is strictly sylvatic [21].

Geographical origin and cytogenetic traits of the *Triatoma* species analysed are detailed in Table 1. These species were selected by their different number of autosomes (18, 20 and 22), male sex systems (XY and X_1_X_2_Y), and differences in the amount and chromosomal location of autosomal heterochromatin [15, 22–25].

**Table 1:** Geographical origin and chromosomal traits of seven *Triatoma* species analysed in the present studyk

Species and male diploid chromosome number (2n)	Geographical origin	%, chromosome location and size of autosomal C-heterochromatin
*T. rubrofasciata* (2n = 22A + X_1_X_2_Y)	Vietnam, Hanoi, Tu Liem district. P. 21°2′48″N, 105°44′54″E	40%; 11 II with C-blocks in both chromosomal ends [22]
*T. dimidiata* (2n = 20A + X_1_X_2_Y)	Guatemala, Jutiapa, Carrizal. D. 14°25′48″N, 89°57′28″W	10%; 10 II with C-dots in both ends [23]
*T. barberi* (2n = 20A + X_1_X_2_Y)	Mexico, Queretaro, La Cueva. P. 20°29′4″N, 100°26′20″W	35%; 10 II with C-blocks in both ends [15]
*T. nitida* (2n = 18A + X_1_X_2_Y)	Guatemala, Quiché, Zacualpa, D. 15°1′34″N, 90°52′42″W	25%; 2 II almost entirely C-heterochromatic [15]
*T. lecticularia* (2n = 20A + XY)	Insectary CDC (Atlanta). Origin: USA, Oklahoma, Walkiria.	30%; 10 II with C-blocks in both ends [15]
*T. infestans* (non-Andean lineage) (2n = 20A + XY)	Argentina, Chaco, Tres Estacas. P. 26°54′30″S, 51°40′23″W	24–30%; 2–4 II with C-blocks in one or both ends [24]
*T.* (*Mepraia*) *spinolai* (2n = 20A + X_1_X_2_Y)	Chile, Metropolitan Region of Santiago, Colina. S. 33°11′53″S, 70°39′42″W	15%; 10 II with C-dots in both ends [25]

*Abbreviations*: *A* autosomes, *D* domestic, *P* peridomestic, *S* sylvatic, *II* bivalents

Genomic DNA (gDNA) probes were made from two *Triatoma* species of the rubrofasciata group: *T. rubrofasciata* collected in Vietnam (Hanoi city, Tu Liem district, Dai Mo commune, Ngoc Truc village) and *T. dimidiata* from Guatemala (Jutiapa, Carrizal). Genomic DNA isolation for probes generation was made from legs of one adult male individual using the NucleoSpin Tissue kit (Macherey-Nagel Co., Düren, Germany). For probe labelling, total genomic DNA was labelled with biotin-16-dUTP (Roche, Mannheim, Germany) using a Nick Translation Kit (Roche, Shanghai, China), following manufacturer’s instructions. To compare the results obtained with these two probes, we applied a third genomic probe (*T. infestans*) that was already used in a previous paper [16] but applied here for the first time on *T. rubrofasciata* chromosomes.

Chromosome preparations for C-banding and GISH analyses were obtained from males. Gonads were removed from live adult insects, fixed in ethanol: glacial acetic acid mixture (3:1) and stored at -20 °C. Squashes were made in a 50% acetic acid drop, coverslips were removed after being frozen in liquid nitrogen and the slides were air dried and then stored at 4 °C. C-banding was performed according to Panzera et al. [24].

In situ hybridizations were carried out as described by Palomeque et al. [26]. Hybridization solutions were prepared to a final concentration of 0.5–2.0 ng probe/ml in 50% formamide. Hybridization was conducted at 37 °C overnight. Fluorescence immunological detection was performed using the avidin-FITC/ anti-avidin-biotin system with two amplification rounds. Slides were mounted with Vectashield (Vector, Burlingame, USA). DAPI in the antifade solution was used to counterstain chromosomes. The hybridized chromosomes were observed and photographed using a BX51 Olympus fluorescence microscope equipped with a CCD camera (Olympus DP70) and merged using the DP Manager software. Hybridization pattern for each species was determined by the chromosomal analyses of at least two individuals.

## Results

**Fig. 1:**
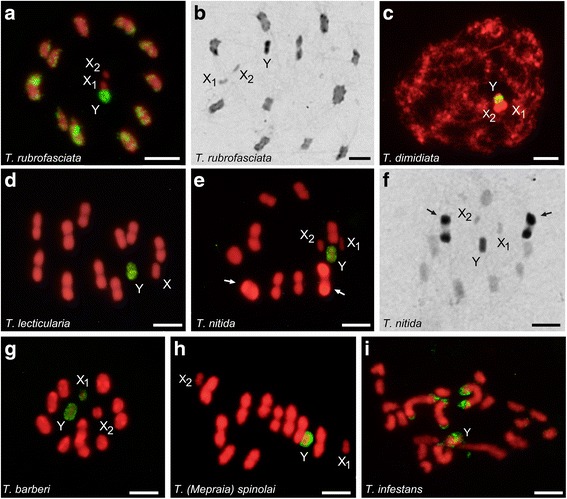
GISH results using *Triatoma rubrofasciata* genomic DNA (gDNA) probe (labelled in yellow-green) on male chromosomes of different *Triatoma* species (labelled in red). **a** Self-GISH on own chromosomes of *T. rubrofasciata* (2n = 22A + X_1_X_2_Y): Second meiotic metaphase. Hybridization signals appear scattered on all chromatin, but the strongest signals are preferably located at the autosomal chromosome ends plus the Y chromosome. Both X chromosomes did not present hybridization signals. **b** *T. rubrofasciata.* First meiotic metaphase (MI) with C-banding. Heterochromatic regions with the same distribution pattern as observed with self-GISH. **c** *T. dimidiata* (2n = 20A + X_1_X_2_Y). Early meiotic prophase. **d** *T. lecticularia* (2n = 20A + XY): MI. **e** *T. nitida* (2n = 18A + X_1_X_2_Y): MI. In (**c**) to (**e**), strong hybridization signals are restricted to the heterochromatic Y chromosome. **f** *T. nitida*: MI with C-banding. The two C-heterochromatic bivalents did not exhibit hybridization signals (compare chromosomes pointed out with arrows in **e** and **f**). **g** *T. barberi* (2n = 20A + X_1_X_2_Y): MI. Y chromosome and one of the X chromosomes (X_1_) appear with strong signals. **h** *T.* (*Mepraia*) *spinolai* (2n = 20A + X_1_X_2_Y): MI. Only the Y chromosome shows hybridization signals. In (**c**) to (**h**), autosomal C-heterochromatic regions appear labelling free. **i** *T. infestans* (2n = 20A + XY): Spermatogonial prometaphase. Strong hybridization signals are observed on the Y chromosome and five autosomes. *Scale-bars*: 5 μm. *Abbreviations*: A, autosomes; MI, metaphase I

**Fig. 2:**
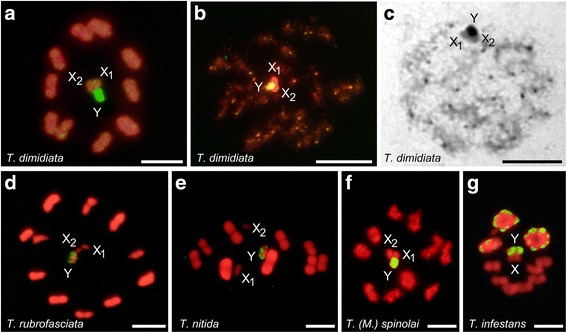
GISH results using *Triatoma dimidiata* genomic DNA (gDNA) probe (labelled in yellow-green) on chromosomes of different *Triatoma* species (labelled in red). **a** Self-GISH on own chromosomes of *T. dimidiata* (2n = 20A + X_1_X_2_Y): Second meiotic metaphase (MII). All chromatin presented scattered hybridization signals, but strong signals were observed only on the Y chromosome. **b** *T. dimidiata*: Early meiotic prophase showing dot hybridization signals at the chromosome ends of all autosomes and on the Y chromosome. **c** *T. dimidiata*: Early meiotic prophase with C-banding. C-Heterochromatin regions with the same distribution pattern as observed with the genomic probe in **b**. **d** *T. rubrofasciata* (2n = 22A + X_1_X_2_Y): MII. **e** *T. nitida* (2n = 18A + X_1_X_2_Y): Metaphase I (MI). **f** *T.* (*Mepraia*) *spinolai* (2n = 20A + X_1_X_2_Y): Diakinesis. In **c**-**e**, the Y chromosome exhibited strong hybridization signals. **g** *T. infestans* (2n = 20A + XY): MI. Strong hybridizations signals were observed on the Y chromosome and chromosomal ends of three bivalents. *Scale-bars*: 5 μm. *Abbreviations*: A, autosomes; MI, metaphase I; MII, metaphase II

**Fig. 3:**
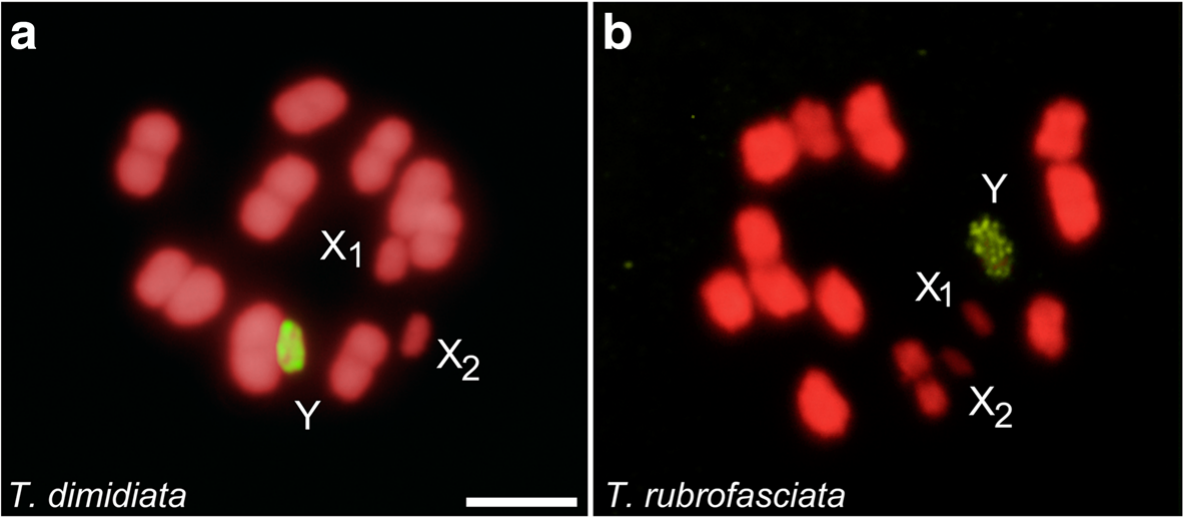
GISH results using *Triatoma infestans* genomic DNA (gDNA) probe (labelled in yellow-green) on metaphase I chromosomes (labelled in red) of *T. dimidiata* (**a**) and *T. rubrofasciata* (**b**). In both species, only the heterochromatic Y chromosome appears with strong hybridization signals, while autosomal heterochromatic regions appear label free. *Scale-bars*: 5 μm

We observed two chromosomal hybridization patterns: (i) very intense hybridization signals concentrated on specific chromosomal regions or particular chromosomes; and (ii) lower intensity hybridization signals dispersed along all chromosomes (Figs. 1, 2 and 3). In some cases, the lower intensity hybridization signals were masked by the DAPI signal in the merged figures. Table 2 summarises the GISH results described below.

**Table 2:** Summary of GISH results using two genomic DNA probes of North American *Triatoma* on the chromosomes of seven *Triatoma* species

Species	*T. rubrofasciata* genomic DNA probe	*T. dimidiata* genomic DNA probe
*T. rubrofasciata*	Self-GISH. All autosomal pairs (11 half-bivalents) with strong hybridization signals in one or both chromosomal ends. Y chromosome intensively and totally labelled (Fig. 1a)	Only Y chromosome (Fig. 2d)
*T. dimidiata*	Only Y chromosome (Fig. 1c)	Self-GISH. All bivalents (10) with strong hybridization signals in both chromosomal ends. Y chromosome intensively and totally labelled (Fig. 2a, b)
*T. lecticularia*	Only Y chromosome (Fig. 1d)	Only Y chromosome.
*T. nitida*	Only Y chromosome (Fig. 1e)	Only Y chromosome (Fig. 2e)
*T. barberi*	Y chromosome plus one X chromosome (Fig. 1g)	Y chromosome plus one X chromosome
*T.* (*Mepraia*) *spinolai*	Only Y chromosome (Fig. 1h)	Only Y chromosome (Fig. 2f)
*T. infestans* (non-Andean lineage)	2–3 autosomal pairs with strong hybridization signals in 1 or 2 chromosomal ends plus the Y chromosome (Fig. 1i)	2–3 bivalents with strong hybridization signals in 1 or 2 chromosomal ends plus the Y chromosome (Fig. 2g)

### *Triatoma rubrofasciata* gDNA probe

Self-GISH results on *T. rubrofasciata* showed that almost all chromatin presented scattered hybridization signals. Strong hybridization signals were observed in C-heterochromatin regions: chromosomal ends of the 11 half-bivalents plus the Y chromosome, but neither X chromosome presented strong hybridizations signals (Fig. 1a). A similar pattern was observed with C-banding (Fig. 1b). Hybridization of all North American *Triatoma* species showed strong hybridization signals only on the Y chromosome (Fig. 1c-g), without labelling on other heterochromatic regions (compare Fig. 1e with f, arrows). In *T. barberi*, in addition to the Y chromosome, one of the two X chromosomes is also labelled (X_1_) (Fig. 1g).

*Triatoma rubrofasciata* gDNA probe on chromosomes of South American *Triatoma* showed differences between the two species analysed. In *T.* (*Mepraia*) *spinolai,* only the Y chromosome exhibited hybridization signals (Fig. 1h). In *T. infestans*, in addition to the Y chromosome, 2 to 3 autosomal pairs showed terminal hybridization signals (Fig. 1i).

### *Triatoma dimidiata* gDNA probe

Self-GISH results on *T. dimidiata* showed strong hybridization signals on the Y chromosome as well as scattered hybridization signals on all chromatin, including both X chromosomes (Fig. 2a). However, on less condensed chromosomes (early meiotic prophase), strong hybridization signals were observed as spots on the chromosomal ends of all autosomes (Fig. 2b). These hybridization signals correspond to the heterochromatic dot regions located on the chromosomal ends observed with C-banding (Fig. 2c). When *T. dimidiata* gDNA probe was employed on chromosomes of *T. rubrofasciata* and other North American *Triatoma* species, only the Y chromosome presented positive hybridization signals (Fig. 2d, e), with the exception of *T. barberi*, which exhibited the same pattern observed with *T. rubrofasciata* probe (one X chromosome also labelled, data not shown). In South American *Triatoma* species, hybridization patterns were the same as the obtained with *T. rubrofasciata* genomic probe, i.e. only on the Y chromosome in *T. spinolai* (Fig. 2f) and 2–3 bivalents plus the Y chromosome in *T. infestans* (Fig. 2e).

### *Triatoma infestans* (non-Andean lineage) gDNA probe

GISH results on *T. dimidiata* (Fig. 3a) and *T. rubrofasciata* (Fig. 3b) chromosomes showed strong hybridization signals only on the Y chromosome, while the autosomal heterochromatic regions remain unmarked.

## Discussion

### GISH technique and DNA repeat sequences

This paper and the previous one [16] show that triatomine GISH probes reveal two hybridization patterns: scattered hybridization signals along almost all chromatin and stronger hybridization signals concentrated on particular chromosomal regions or on the entire chromosomes (Figs. 1, 2 and 3). The stronger signals could be caused by satellite DNA families clustered in long tandem arrays as was seen using specific satDNA probes [27]. Meanwhile, the scattered signals might be produced by small amounts of satellite DNA families [27, 28], or by transposable genetic elements, as has been observed in other insects [29, 30].

From GISH results obtained here (Table 2, Figs. 1, 2 and 3) we can draw the following assumptions. First, in the two Self-GISH or Auto-GISH analyses, i.e. *T. rubrofasciata* genomic probe on *T. rubrofasciata* chromosomes and *T. dimidiata* gDNA probe on *T. dimidiata* chromosomes, the strong hybridization signals coincide exactly with the C-heterochromatic regions described for each species (compare Fig. 1a with b, and Fig. 2b with c, respectively). However, when we perform genomic cross-hybridizations, i.e. *T. rubrofasciata* gDNA on *T. dimidiata* chromosomes (Fig. 1c) and vice versa (Fig. 2d), only strong signals are detected on the Y chromosome, leaving the C-heterochromatic autosomal regions unmarked. This reveals that the heterochromatic regions of *T. rubrofasciata* and *T. dimidiata* are constituted by different classes of satellite DNA families, which appear undifferentiated with C-banding and fluorescence staining [15]. This same conclusion can be extended to the other North American *Triatoma* (*T. lecticularia, T. nitida* and *T. barberi*) (Fig. 1d-g; Fig. 2e), which have a different amount and chromosomal localization of heterochromatin (Table 1). This evidence is conclusive in showing that, at least in the species here analysed, chromosome diversification in North American *Triatoma* involved a differential amplification of diverse types of autosomal repeated DNA sequences. This is very different from what has been described for the infestans subcomplex species (*T. infestans*, *T. platensis* and *T. delpontei*), where these closely related species shared their repeated DNA sequences [16].

Secondly, the hybridization patterns of both genomic probes on *T. infestans* chromosomes showed strong signals on autosomal heterochromatic regions (Figs. 1i and 2g). These results confirm those obtained with South American *Triatoma* genomic probes, revealing that in the infestans subcomplex the repeated sequences of the Y chromosome are also present on the autosomal heterochromatic regions [16]. Genomic hybridizations using *T. infestans* gDNA probe on *T. dimidiata* [16] and *T. rubrofasciata* chromosomes (Fig. 3a, b, respectively) showed strong hybridization signals only on the Y chromosome. These results reveal that autosomal heterochromatic regions of *T. dimidiata* and *T. rubrofasciata* are constituted by different DNA repeats, most probably satDNA families, which are also different from what was observed for the Y chromosome.

### Evolution of sex chromosomes

In triatomines, sex chromosomes are very well differentiated from autosomes by their distinct behaviour during meiosis. During male meiotic division, X and Y chromosomes are asynaptic and achiasmatic, showing an inverted meiosis [31, 32]. Three male sex systems have been reported in Triatominae subfamily: XY, X_1_X_2_Y, and X_1_X_2_X_3_Y. The first system is considered ancestral so that the multiple Xs are due to fission processes of the original X [33]. Multiple sex chromosomes have been reported in several triatomine genera including *Triatoma* species from North and South America [10]. Given that these multiple sex systems appear in triatomine species that are not closely related, it is probable that the fission processes of the X chromosome have occurred several times during the evolution of this group.

Knowledge about the DNA sequences that composed sex chromosomes in heteropteran species is very limited. Recently, Gallo et al. [34] have analysed the repetitive DNA composition of the Y and X chromosomes in several species of the giant water bugs (Heteroptera: Belostomatidae). These authors showed that repetitive DNA was absent on the Y chromosome while X chromosomes and autosomes shared repetitive sequences. Our previous results obtained by GISH [16], fluorescent banding [15], X chromosome microdissection [17] and the results displayed here support the idea that the X and Y chromosomes in triatomines presented substantial differences in their DNA composition. All of these studies pointed out that the Y chromosome is extremely conserved among all Triatomini species. It is C-heterochromatic and DAPI positive [10, 15], very different to that observed in Belostomatidae. This study confirms that the Y chromosome in North and South American *Triatoma* share repeated DNA sequences (Figs. 1, 2 and 3). Very recently we proved that the Y chromosome in *T. infestans* is constituted by at least two satDNA families [35]. The sequence conservation of the Y chromosome in a speciose and diverse insect group is very uncommon, and probably represents an ancestral character of Triatomini tribe, such as suggested by Pita et al. [16].

By contrast, the X chromosome is euchromatic, without fluorescence staining [15] but with dispersed repeated sequences similar to those observed in euchromatic autosomal regions [17, 35]. Occasionally in some species, like *T. barberi* (Fig. 1g) and the infestans subcomplex species (*T. infestans* Andean lineage*, T. platensis* and *T. delpontei*) [16], the X chromosomes have heterochromatic regions similar to those observed in the Y chromosome. Probably this sequence homology between the X and Y chromosomes are due to repeated sequences transferred from Y chromosome to the X chromosome. Since *T. barberi* and the infestans subcomplex species are evolutionarily distant species, this transference appears to have occurred several times in the evolutionary history of *Triatoma*, perhaps as a product of secondary differentiation processes. The occurrence of shared sequences between X and Y chromosomes have also been seen with the major ribosomal genes in several *Triatoma* and *Rhodnius* species [12–14, 36]. The existence of shared repeated sequences between the X and the Y chromosomes is not consistent with the accepted notion that the sex chromosomes of Heteroptera do not exchange sequences [32]. Mobilization of repeated sequences between sex chromosomes without recombination or chromosomal pairing could be due to transposition events, as has been suggested in ants [37] and triatomines [13].

Another alternative hypothesis to explain the sequence homology between the Y chromosome and one of the X chromosomes in *T. barberi* is that this heterochromatic X_1_ chromosome was originated by fission from the Y chromosome, rather than fissions of an ancestral X as is currently accepted. Considering that the X_1_X_2_Y sex system is the only one present in 22 North American *Triatoma* species currently analysed (except *T. lecticularia* with XY) and that the X chromosome is almost always euchromatic [10], this hypothesis seems improbable but cannot be ruled out.

## Conclusions

Our GISH results allow us to draw the following conclusions: (i) Y chromosome repeated sequences seem to be the only ones shared among all *Triatoma* species, and even in the whole Triatomini tribe (which also involves six other genera); (ii) the five North American *TriatoXma* species here analysed did not share their autosomal repeated DNA sequences, in spite of their close evolutionary relationships; and (iii) this divergence in the molecular composition of autosomal repetitive DNA is also observed between North and South American *Triatoma*. These results suggest that autosomal heterochromatin in triatomines consists of different classes of repeated sequences, probably satDNA, so that during the speciation of this genus amplifications of different repeated DNA families have occurred. Molecular characterization of such repetitive DNA families seems to be an appropriate approach to identify the diversification mechanisms of the repeated sequences and to infer evolutionary relationships in triatomines.
